# Utility of transthoracic impedance and novel algorithm for sleep apnea screening in pacemaker patient

**DOI:** 10.1007/s11325-018-1755-y

**Published:** 2018-11-24

**Authors:** RuoHan Chen, KePing Chen, Yan Dai, Shu Zhang

**Affiliations:** Center of Arrhythmia, Fuwai Hospital, Chinese Academy of Medical Sciences, Peking Union Medical College, Beilishi Road 167#, Xicheng Qu, Beijing, 100037 China

**Keywords:** Pacemakers, Polysomnography, Sleep apnea syndromes

## Abstract

**Background:**

Pacing patients was revealed with a high prevalence of sleep disorder, but mostly undiagnosed. The pacemaker with transthoracic impedance sensor and novel algorithm could identify sleep apnea (SA) event. This study aimed to evaluate accuracy of pacemaker in sleep apnea diagnosis.

**Methods:**

This is a prospective study which enrolled patients implanted with pacemakers integrated with transthoracic impedance sensor and SA-identified algorithm (AP Scan). All patients underwent a polysomnography (PSG). The apnea and hypopnea index (AHI) of the PSG (PSG-AHI) and the respiratory disturbance index (RDI) of the pacemaker (PM-RDI) were recorded on the same night. The correlation between two methods was evaluated by the kappa coefficient, receiver operating characteristic (ROC) curves, and Bland and Altman statistics.

**Results:**

Sixty-four patients were enrolled, who had never been diagnosed with SAS or underwent PSG exam. After PSG examination, 76.4% patients were diagnosed as combining with SA (20% severe, 18.2% moderate, and 38.2% mild). RDI calculated by PM has a strong positive correlation with PSG-AHI (*r* = 0.76, *p* < 0.001, 95% CI 0.61–0.85). The optimal cutoff value of PM-RDI for advanced SAS (PSG-AHI ≥ 15) diagnosis was 26, with AUC of 0.89 (95% CI 0.77 to 0.96, *p* < 0.001). The best cutoff value for severe SA (PSG-AHI ≥ 30) identification was 41, with a sensitivity of 81.6%, a specificity of 88.6%.

**Conclusions:**

Pacemaker patients present a high prevalence of undiagnosed SA. Detection of SA by pacemaker is feasible and accurate in SA screening and monitoring.

## Introduction

Sleep apnea syndrome (SAS) is a sleep disorder characterized by cessation in breathing. Moderate-to-severe SA has been described in up to 23% women and 49% man between ages 30 and 60 [1]. Cardiovascular disease patients have a higher prevalence of SA than the general population and combining with SA links with negative cardiovascular outcomes, such as hypertension [2], heart failure progression [3, 4], and cardiac arrhythmias. Polysomnography (PSG) was the “golden standard” for SAS diagnosis. But the expense and unavailability of PSG greatly limited its screen for SAS widely. Despite increased awareness, SAS remains underdiagnosed [5].

Previous study has shown that the transthoracic impedance measured by pacemaker minute ventilation sensors is closely correlated with the tidal volume [6]. Therefore, the impedance might be used to detect the disturbances of ventilation during sleep. Recently, some pacemakers with ventilation sensor and special algorithms have been reported to be capable of screening sleep apnea [7, 8]. The aim of this study was to appraise the accuracy of an advanced algorithm (Apnea Scan [ApScan] algorithm) in detecting severe SA in unselected pacing candidate.

## Methods

### Study design

This prospective singly-center study has been entirely carried out at the Fuwai Hospital (Beijing, China). The patients who underwent implantation of a Vitalio J237 or J274 (Boston Scientific, St. Paul, Minnesota, USA) from August 2016 to December 2017 were enrolled in the study, despite of whether the patients have SA symptoms or diagnosis. Patients were enrolled consecutively. All the patients have the indication for pacemaker implantation according to the available guidelines [9]. The choice of leads and the position of leads’ placement were decided by the operators. Information including demography, underline heart and pulmonary disease, pacing indication, current medications, left ventricular ejection fraction (LVEF), and Epworth scores were obtained from each patient. This study has been approved by local ethics committees, and all enrolled patients were required to sign an informed consent.

### Pacemaker follow-up

Three follow-ups were scheduled: the second day after implantation, prehospital discharge, and 3 months after operation. The routine checks of the devices and interrogations of the store data were performed during each follow-up. Between the first and the second visits, patients spent one night to undergo PSG. The pacemaker data obtained by ApScan algorithm were interrogated in the morning after PSG.

### Epworth sleeping scale

Before pacemaker implantation, all patients performed the Epworth Sleeping Scale (ESS). ESS is a standardized questionnaire assessing propensity for sleepiness in eight common situations (e.g., doing some reading, waiting for the traffic lights). The likelihood of dozing was graded from a scale of 0 (would never doze) to 3 (high chance of dozing) for each situation. The ESS score is the sum of the eight items [10]. A score of equal to or greater than 10 is recognized as an indicator excessive daytime somnolence.

### PSG measurements

Overnight PSG was performed and interpreted by a sleep specialist who was blinded to the PM-RDI data. PSG examination included electroencephalogram (EEG) for sleep analysis and electromyogram for chest and abdominal respiratory movement, oral-nasal airflow, and arterial oxyhemoglobin saturation. Hypopnea event is a decline of tidal volume at least 30% from baseline lasting 10 s or long. Apnea event is a suspension of oral-nasal airflow lasting 10 s or more. The PSG-AHI was the average number of apnea and hypopnea events per hour during sleep time. The severity of SA was graded by the AHI scores [11], that is, PSG-AHI ≥ 30: severe SA; 15 < PSG-AHI < 30: moderate SA; 5 <PSG-AHI < 15: mild SA. The categorization of SA was defined by the following: obstructive sleep apnea (OSA), characterized the pauses in nasal airflow but inhalation movement continues; central sleep apnea (CSA) is not only the nasal airflow but also the chest and abdominal movements are diminished or absent; and mixed sleep apnea (MSA) is a combination of the OSA and CSA.

### Transthoracic impedance and ApScan algorithm

The pacemaker calculated the impedance from the tip of pacing lead to the can of pacemaker. Specifically, a subthreshold pulse (320 μA, 19.5 μs, 20 Hz) is delivered from the can to RV coil, and voltage is measured from the can to RV tip, and transthoracic impedance was calculated by current and voltage. An appropriate filtering was applied to avoid the influence came from cardiac contractions, body posture, and respiratory movement. Transthoracic impedance fluctuated regularly with respiratory movement. During inspiration, the transthoracic impedance is high, and during expiration it is low, so that it can be optimized to recognize respiratory cycle. The differential of transthoracic impedance in a cycle reflected the tidal volume and could be utilized to estimate the variation in tidal volume. According to the AP Scan algorithm, respiratory disturbance event was defined as a decline of tidal volume at least 26% from the baseline or breathe suspended at least 10 s or more [12]. The sleep time was set individually according to the patient sleep habit, usually, 11 pm–6 am, but at least 7 h for RDI calculation. Respiratory disturbance index (PM-RDI) could be evaluated as the average number of respiratory disturbance events per sleeping hour. When interrogated, day to day PM-RDI could display not only by numbers but also by curve, which helps the physician calculates the burden of severe sleep apnea.

### Statistical analysis

Categorical variables were presented as the number and percentage. Continuous variables were presented as mean ± SD. Agreement between two methods was analyzed by the Bland-Altman method and Cronbach’s alpha. Pearson’s correlation coefficient was applied to evaluate the correlation between the PM-RDI and the PSG-AHI. The accuracy identification of moderate/severe SA by pacemaker was appraised by receiver operating characteristic (ROC) curve. The sensitivity, specificity, positive predictive value (PPV), and negative predictive value (NPV) for the optimal cutoff values were calculated. All statistical analyses were performed by SPSS 22.0 package program. A *p* value < 0.05 was considered significant difference.

## Results

### Study population

A total 64 patients were enrolled. Their mean age was 67.1 ± 9.8 years, and mean BMI was 24.0 ± 4.2 kg/m^2^; most of patients were male 40 (62.3%). No patient had ever been diagnosed with SA or underwent the PSG examination before this study.

Eight patients refused or were not tolerant of PSG examination; one patient lost the PM-RDI data. Fifty-five patients completed PSG test and had corresponding RDI data. Their data were analyzed for SA diagnosis and performances of ApScan algorithms. Detailed information was summarized in Table 1.

**Table 1:** Demographic and clinical characteristics

Characteristics	Total (*N* = 55)	No or mild SA	Moderate or severe SA	
		(*N* = 34)	(*N* = 21)	*p*
Male	34 (61.9%)	20 (58.8%)	14 (66.7%)	0.77
Age (year)	67.1 ± 9.8	66.5 ± 10.0	68.6 ± 10.3	0.44
BMI (Kg/m^2^)	24.0 ± 4.2	24.5 ± 4.4	23.4 ± 3.6	0.38
Smoker	17 (30.9%)	9 (26.5%)	8 (38.1%)	0.38
PM indication				0.382
SSS	54	34	20	
AVB	1	0	1	
Comorbidities				
Hypertension	33 (60%)	20 (58.8%)	13 (61.9%)	0.52
Diabetes	7 (12.7%)	2 (5.9%)	5 (23.8%)	0.09
Coronary heart disease	16 (29.1%)	7 (20.6%)	9 (42.9%)	0.12
Atrial fibrillation	27 (49.1%)	14 (41.2%)	13 (61.9%)	0.17
LVEDD (mm)	47.4 ± 3.1	47.3 ± 3.3	47.4 ± 2.5	0.83
LVEF (%)	63.7 ± 16.1	64.3 ± 4.3	63.5 ± 3.0	0.46
ESS score (> 10)	11 (20%)	3 (8.8%)	8 (38%)	0.008
PSG-AHI (events/h)	17.4 ± 16.5	7.4 ± 4.2	33.9 ± 15.4	< 0.001
PM-RDI (events/h)	32.3 ± 16.1	23.5 ± 11.9	44.4 ± 13.4	< 0.001

*BMI*, body mass index; *LVEDD*, left ventricular end-diastolic dimension; LVEF, left ventricular ejection fraction; *PSG-AHI*, apnea-hypopnea index evaluated by PSG; *ESS*, Epworth Sleeping Scale; *PM-RDI*, respiratory disturbance index evaluated by the ApScan algorithm

### Prevalence of sleep apnea

The average PSG-AHI is 17.4 ± 16.5 events/h (ranged from 1.1 to 70.8 events/h). According to guideline, 21 (38.2%) patients met a criterion of mild SA (5 ≤ PSG-AHI < 15), 10 (18.2%) patients were moderate SA (15 ≤ PSG-AHI <30 h), and 11 (20.0%) were severe SA (PSG-AHI > 30). Among the moderate and severe SA patients, 14 (66.7%) patients were obstructive SA, 5 (23.8%) were hypopnea, 1 (4.8%) central SA, and 1 (4.8%) mixed SA.

### Correlation of PSG-AHI

**Fig. 1:**
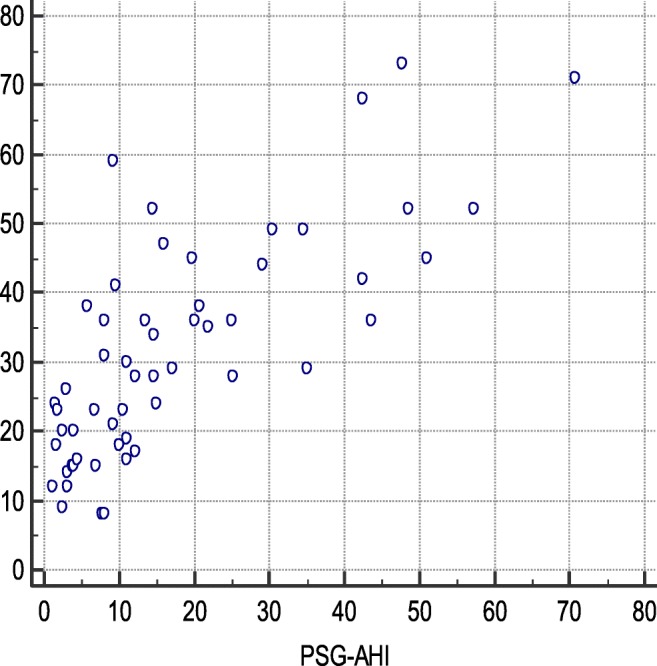
Scatter plot. PM-RDI respiratory disturbance index, calculated by the ApScan algorithm; PSG-AHI apnea-hypopnea index, came from PSG

**Fig. 2:**
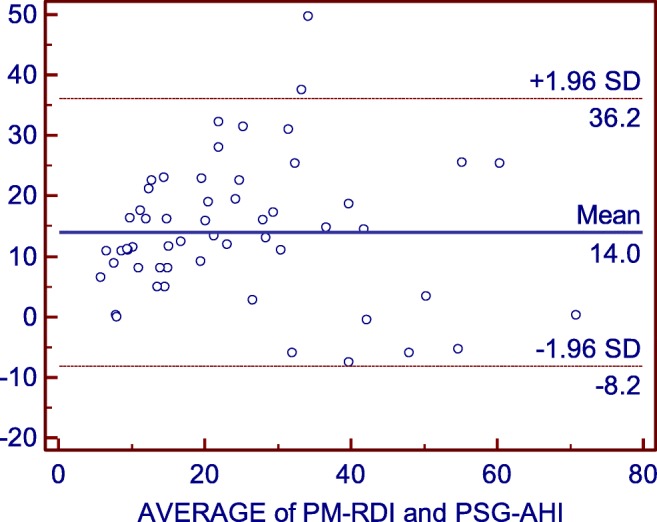
Bland-Altman plot. (*x* axis, the mean of the PM-RDI and PSG-AHI; *y* axis, the difference between the PSG-AHI and the PM-RDI

**Fig. 3:**
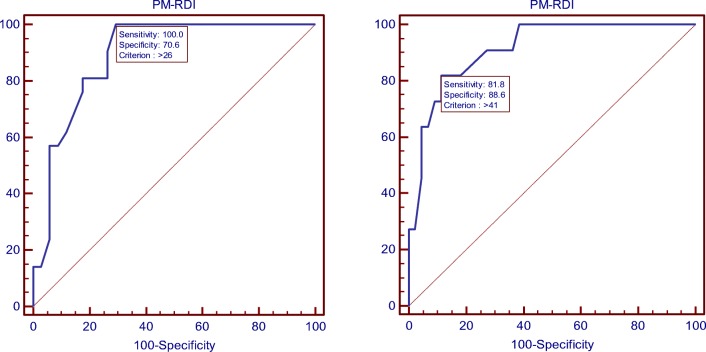
The ROC curve for the detection of SA at PSG-AHI ≥ 30 (right) and PSG-AHI ≥ 15 (left)

There was a strong correlation between the PSG-AHI and the PM-RDI (*r* = 0.76, 95% CI 0.61 to 0.85 *p* < 0.001) (Fig. 1). The reliability between PM-RDI and PSG-AHI was acceptable with the Cronbach’s alpha equaling to 0.86. The mean difference between the PM-RDI and the PSG-AHI was 14.0 ± 22.2 events/h by the Bland-Altman test (Fig. 2). According to ROC analysis, ApScan algorithm detection with moderate to severe SA (PSG-AHI ≥ 15events/h) was best achieved with AUC of 0.89 (95% CI 0.77 to 0.96, *p* < 0.001), the cutoff value was 26 events/h. The accuracy of the ApScan algorithm to detect severe SA (PSG-AHI ≥ 30) was also statistically significant with an AUC of 0.91 (95% CI 0.80 to 0.97, *p* < 0.001); an optimal PM-RDI cutoff value for this was 41 (Fig. 3). Due to the good linear correlation between the PSG-AHI and the PM-RDI and the similar diagnosis in the severity of SA by PM and PSG, which explains the delta between moderate SA and severe SA by two measuring methods are same; both were 15 events/h. Table 2 described the best cut-off values of PM-RDI, including sensitivity, specificity, and positive and negative predictive values.

**Table 2:** Effects of PM-RDI cutoffs on risk stratification performance

Index	PSG-AHI ≥ 15	PSG-AHI ≥ 30
	PM-RDI ≥ 26	PM-RDI ≥ 41
Sensitivity (% [95% CI])	100.0 (83.9–100.0)	82.1 (48.2–97.7)
Specificity (% [95% CI])	70.6 (52.5–84.9)	88.6 (75.4–96.2)
Positive predictive value (% [95% CI])	67.7 (48.3–83.5)	64.3 (35.1–87.2)
Negative predictive value (% [95% CI])	100 (85.8–100.0)	95.1 (83.3–99.4)
Positive likelihood ratio (95% CI)	3.4 (2.7–4.2)	7.2 5.3–9.7)
Negative likelihood ratio (95% CI)	0	0.21 (0.05–0.9)

## Discussion

This study appraised the accuracy of AP Scan algorithm in SA screening. First, we reported a high prevalence rate of 76.4% for SA among pacemaker patients, which included 38.2% of the patients exhibiting moderate and severe SA. The prevalence of SA in this study is consistent with previous studies [8, 12] (from 60 to 78%). But, the prevalence of moderate and severe SAS was relatively lower; previously, studies were about 50%, which may because the patients in this study were younger (67.1 ± 9.8 vs 73.8 ± 10.1). Among the advance SA (AHI ≥ 15 events/h) patients, obstructive SA (66.7%) and hypopnea (23.8%) accounted for more than 90%. The correlation between PM-RDI and PSG-AHI was 0.76 (*p <* 0.001), and the bias of AHI was 14.0 ± 22.2/h. The Cronbach’s alpha between PM-RDI and PSG-AHI was 0.86; this result is not inferior than other home-based PSG recording systems [13, 14]. These proved transthoracic impedance measurement together with AP Scan algorithm could be used to screen SA and monitor treatment effects in pacemaker patient. The result showed that an PM-RDI of 26 events/h is the optimal cutoff value for advance SA (AHI ≥ 15events/h) diagnosis, with a specificity of 70.6%, a sensitivity of 100%. The best PM-RDI cutoff value for severe SA diagnosis was 41 events/h; sensitivity and specificity were 81.6% and 88.6%, respectively.

Sleep apnea is one of the most common comorbidities in patients with cardiovascular disease. The PSG has been accepted as the “gold standard”for SA diagnosis, and the PSG-AHI score is used not only to diagnose SA but also to assess the severity. However, expensive and time consuming together with the limited available of sleep lab makes PSG impracticable for many patients. Previous studies revealed SA prevalence in pacemaker patients is high (59%), but mostly undiagnosed. In our study, none patients had ever been diagnosed by SA or undertaken PSG before PM implantation. But accurately, 76% patients combined with SA and more than one third patients were with advance SA. As a result, an alternative, reliable, and more convenient option could greatly improve SA detection. Some portable and home base and monitor became increasingly attractive.

Nowadays, almost all pacemaker could provide the rate adaptive pacing by combining with different kinds of sensors. Among these sensors, minute volume sensor could detect the respiratory rate and tidal volume by calculating transthoracic impedance [15]. This function has been used to not only rate adaptive pacing, but also detect cardiac decompensations [16]. More recently, some researchers have proved that change of respiratory and tidal volume could also be used to detect SA [17]. Compared with PSG, the sensitivity of SA diagnosis by PM was 75~89%, and the specificity was 85~94%. With the novel algorithm, new generation pacemaker could provide the information about SA becoming increasingly attractive. Our study demonstrated PM-RDI had a good correlation in SA diagnosis. The optimal PM-RDI cutoff values for moderate and severe SA were 26 events/h and 41 events/h. Compared with other studies, it was relatively higher. This difference may due to SA definition which varies between different manufacturers. Previously, studies were carried out almost all in Sorin (Paris, France) devices. In this device, SA event was defined by breathing cessation for > 10 s or tidal volume reduced by ≥ 50% for more than 10 s. The pacemaker in our study is from Boston Scientific (Minnesota, USA); the apnoeas defined by AP Scan algorithm was breathing suspended for 10 s or more; hypopnoeas was tidal volume declined 26% of the baseline average tidal volume for > 10 s. We can see these two different devices have different criteria for hypopnoeas event. This may explain different diagnosis sensitivity and cutoff value in this study.

The change in transthoracic impedance could reflect to the change of tidal volumes, but the ventilation is decreased in both CSA and OSA. Pacemaker algorithm could not reveal the cessation of thoracic or abdominal movement, so the obstructive and central events cannot be differentiated by PM algorithms. But the purpose of this function in our opinion is not to substitute the PSG, but to screen SA in pacing patient in whom SA was seriously underdiagnosed. This also explained why nowadays algorithm is not so strict compared with previous ones. As for patient management, a further investigation with PSG is still needed. Due to lack of electroencephalography information, pacemaker could not distinguish sleep and awakening times. ApScan algorithm predefines a core sleep time; PM-RDI was calculated only during the sleep time. In this study, the sleep period set to “23 pm–6 am”. This may produce a systemic error. In real-life scenarios, the sleep time could be set more individually according to patient sleep diaries.

A special aspect of the ApScan algorithm is that it could provide convenient way to screen out SA in PM patients. In our study, nine patients were excluded. Among them, eight were due to lack of PSG data (4 refused PSG, 4 failed to have a result). Only one patient was excluded due to lack of ApScan data. The pacemaker algorithm was seemed to be more applicable. In addition, ability to continuously monitor SA means the possibility of make a diagnosis early and therefore initiating appropriate therapy in time. The last but not the least, the relationship among SA and arrhythmias gained considerable interest. Pacemaker could record the burden of arrhythmia and SA day by day for a period of time. And this will allow a prospective exploration of the relationship between these two clinic events.

In conclusion, SA is highly prevalent in pacemaker patients. Screening for SA with transthoracic impedance and ApScan algorithm may facilitate early diagnosis and timely treatment of SA in pacemaker patients, and provide long-term tendency on SA as well.
